# The validity of the AEON™ stapler in gastrointestinal surgery: early clinical experience and translational rationale

**DOI:** 10.3389/fonc.2026.1777615

**Published:** 2026-04-21

**Authors:** Aali J. Sheen

**Affiliations:** 1 Spire Hospital, Manchester, United Kingdom; 2 Faculty of Biology, Medicine and Health, The University of Manchester, Manchester, United Kingdom

**Keywords:** AEON™ stapler, bariatric surgery, gastrointestinal stapling, haemostasis, hepatopancreatobiliary surgery, surgical innovation

## Abstract

**Background/need:**

Gastrointestinal stapling continues to be limited by bleeding, variable compression and leak-related morbidity, especially in bariatric and hepatopancreatobiliary (HPB) surgery. The AEON™ stapler was developed to improve uniformity of compression and staple formation.

**Methodology and device description:**

AEON™ uses an S3 (sustained, smooth and stable) compression mechanism to reduce tissue shear and optimise B-shaped staple formation. Evidence comes from a randomised controlled trial in sleeve gastrectomy, a large bariatric cohort and observational HPB series.

**Preliminary results:**

Across 1, 848 bariatric cases, only one staple-line leak occurred, and 428 consecutive AEON™ cases had no stapler-related complications. A sleeve gastrectomy trial showed significantly reduced intraoperative bleeding. In a 250-patient comparison, reinforcement was required in 16.8% of AEON™ cases versus 100% using a tri-staple device. Distal pancreatectomy with AEON™ showed a pancreatic fistula rate of 20% compared with 65% for traditional staplers, and major hepatectomy was completed without transfusion in any AEON™ case.

**Current status:**

AEON™ is in active clinical use with early results supporting improved haemostasis and postoperative outcomes. Larger prospective studies are underway.

## Introduction

Although stapling systems have evolved significantly, staple-line bleeding and leaks continue to affect outcomes in gastrointestinal surgery. Bariatric operations demand reliable staple formation due to thick gastric tissue, whereas hepatopancreatobiliatry (HPB) procedures, such as distal pancreatectomy and major hepatectomy, challenge staplers with delicate, highly vascular parenchyma. The AEON™ stapler was engineered to address these limitations by improving the compression profile and stabilising tissue before firing (1).

This review is a narrative synthesis of currently available evidence relating to the AEON™ stapler in gastrointestinal surgery. A structured literature search was performed using PubMed and MEDLINE databases for studies published between 2015 and 2025. Search terms included “AEON stapler”, “gastrointestinal stapling”, “bariatric surgery”, and “hepatopancreatobiliary surgery”.

Eligible studies included randomised controlled trials, comparative cohort studies, and observational case series reporting clinical outcomes associated with AEON™ or comparable stapling platforms. Given the limited number of studies and the emerging nature of the technology, a formal meta-analysis was not performed. Instead, findings are presented descriptively with consideration of study design and inherent limitations.

## Technological innovation and design philosophy

### Device principles

**Figure 1:**
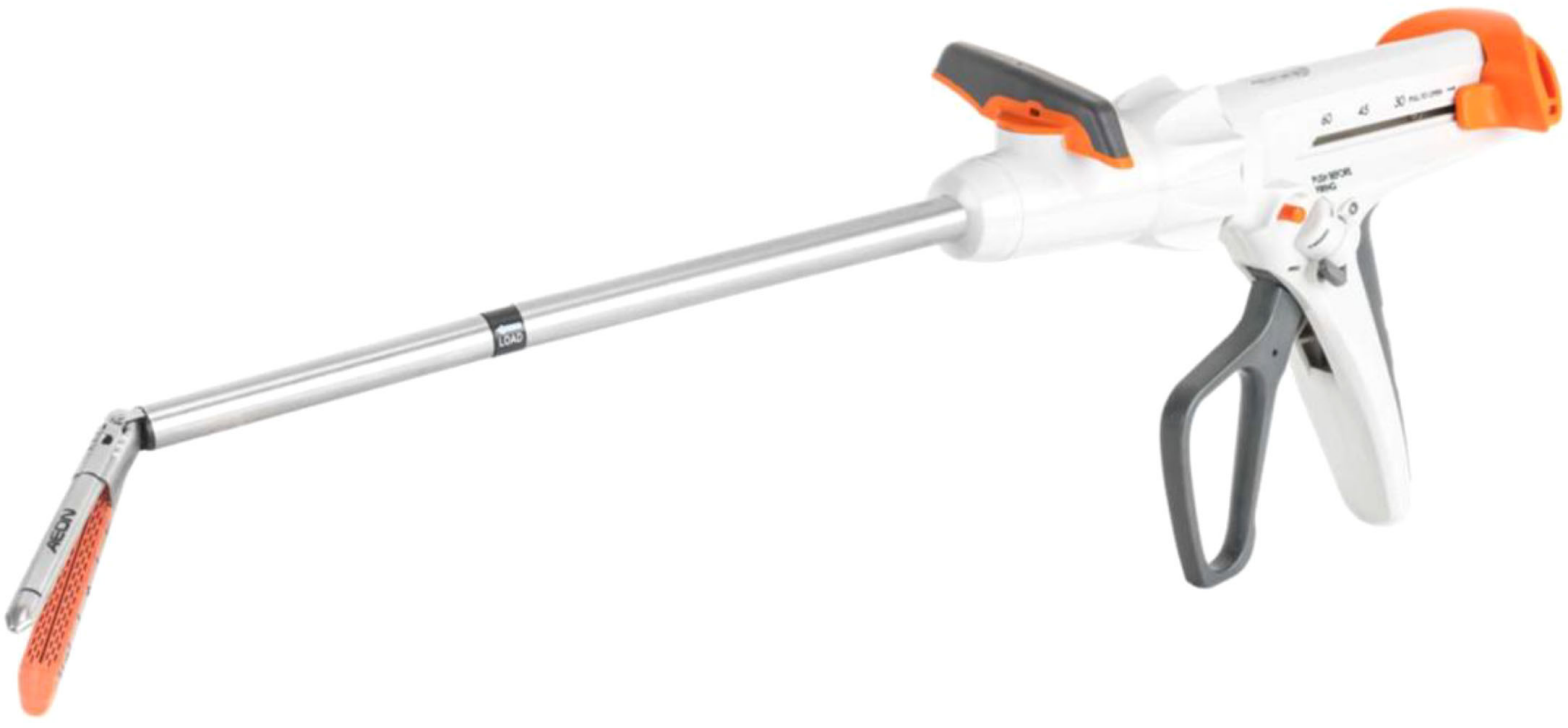
AEON endostapler - the surgical stapler is comprised of two components which work together to deploy the staple line. The first is the handle which is ergonomically designed so that the surgeon can comfortably hold the stapler and feel the tissue. The second component is the reload which contains the single-use titanium staples and blade and is designed for creation of the staple line.

**Figure 2:**
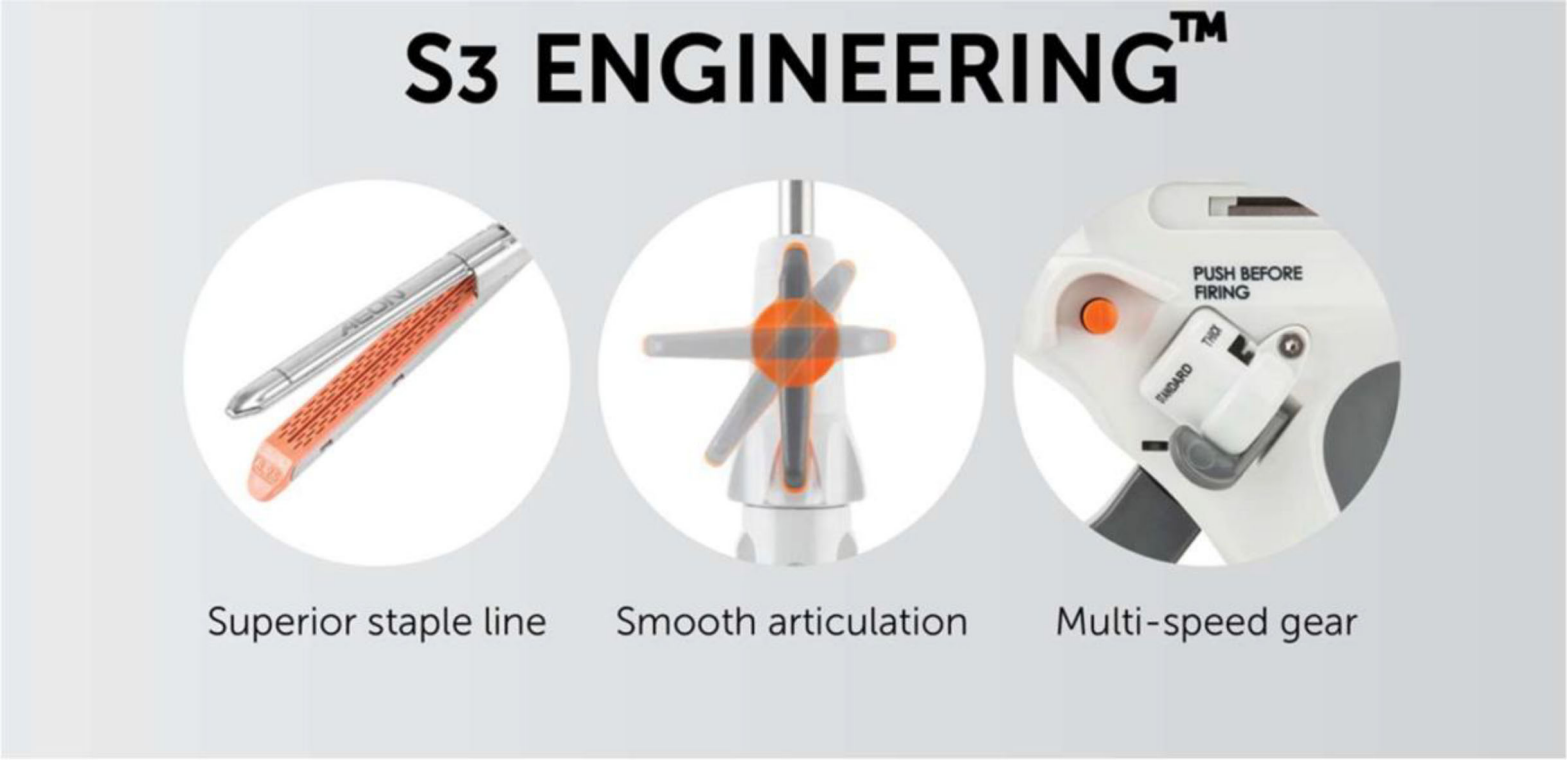
S3 engineering incorporates the three key components of the stapler designed to help create a staple line which is both secure and haemostatic.

AEON™’ S3 compression sequence, sustained, smooth and stable compression, allows tissue to thin uniformly before staple formation, reducing shear injury and producing consistent staple geometry. Surgeons report improved control in deep operative fields, with staple lines exhibiting predictable spacing and tissue incorporation, features designed to enhance haemostasis and decrease leak risk (**Figures 1**, **2**).

### Translational performance in bariatric surgery

The largest evaluation of AEON™ and its immediate predecessor includes 1, 848 laparoscopic bariatric operations (2). These comprise 1, 359 sleeve gastrectomies, 425 Roux-en-Y gastric bypass procedures and 64 single-anastomosis duodeno-ileostomies. Only one staple-line leak occurred across the entire series, and this arose in the comparator-device period. Once AEON™ became established, 428 consecutive procedures were completed without any stapler-related complications, reflecting both device consistency and increasing surgical familiarity.

In the randomised controlled trial assessing sleeve gastrectomy (3), use of the AEON™-based platform resulted in significantly improved haemostasis. Surgeons recorded lower bleeding scores along the staple line, particularly at the gastric fundus where vascularity and tissue thickness pose challenges. Importantly, this enhancement in haemostasis did not increase postoperative leak rates or complication rates.

A further comparative series of 250 sleeve gastrectomies directly compared AEON™ with a widely used tri-staple device (4). Clinical outcomes were equivalent, and no staple-line leaks occurred in either group. However, reinforcement material was applied in 100% of tri-staple cases, whereas only 16.8% of AEON™ cases required reinforcement. This finding is notable because the AEON™ cohort contained proportionally more patients with metabolic comorbidities, including diabetes mellitus. Despite a minor increase in early operative time during AEON™ adoption, the length of stay was shorter, indicating efficient recovery and stable staple-line performance.

### Innovation in HPB surgery

Distal pancreatectomy represents one of the most technically demanding stapling applications due to the risk of postoperative pancreatic fistula (POPF). In a 58-patient cohort comparing AEON™ with traditional staplers (5), POPF occurred in 20% of AEON™ operations compared with 65% of those using other platforms. Biochemical severity reflected this difference, with postoperative day-3 drain lipase concentrations averaging 446 U/L in the AEON™ group compared with 4, 208 U/L in the comparator group. Patients treated with AEON™ had a mean hospital stay of approximately six days, compared with nearly nine days for those treated with other staplers. No severe fistulas, returns to theatre or deaths occurred in AEON™ cases.

Major hepatectomy places additional demands on a stapling device due to the need for secure vascular inflow and outflow control. In a series evaluating AEON™ in laparoscopic major liver resection (6), operative time, parenchymal transection time and postoperative complication rates were equivalent to those of established staplers. A key distinction was the absence of blood transfusion in all AEON™ cases, whereas transfusion was required in a proportion of procedures using comparison devices. This suggests robust vascular sealing and effective staple formation during high-risk transection (7).

### Safety, adoption, and learning curve

From an innovation perspective, a favourable safety profile and short learning curve are essential for successful clinical adoption. Across reported bariatric and HPB series, AEON™ demonstrates no increase in major complications and appears readily adoptable by experienced surgeons once basic familiarity is achieved (2–7). As with all stapling technologies, outcomes remain dependent on sound surgical technique and appropriate cartridge selection.

## Limitations and study heterogeneity

The current evidence base is heterogeneous, comprising a mixture of randomised trials, retrospective cohorts, and observational series with varying sample sizes and endpoints. Differences in patient populations, operative techniques, and definitions of outcomes such as bleeding and leak limit direct comparability between studies.

Furthermore, most available data are derived from early clinical experience and are subject to selection bias and institutional expertise. As such, while the findings are encouraging, they should be interpreted with caution until validated by larger, multicentre prospective studies.

## Conclusion

Across bariatric and HPB domains, early evidence demonstrates that AEON™ provides consistent mechanical performance with tangible clinical advantages. The extremely low leak rate in bariatric surgery (2), improved haemostasis in randomised comparison (3), reduced requirement for reinforcement (4), markedly lower postoperative pancreatic fistula rates in distal pancreatectomy (5), and the elimination of transfusion in major hepatectomy (6) collectively support AEON™ as a promising innovation in gastrointestinal stapling. Further prospective evaluation is warranted, but current findings strongly support its translational validity.
